# The search for blood biomarkers that indicate risk of adverse neurodevelopmental outcomes in fetal growth restriction

**DOI:** 10.3389/fped.2024.1396102

**Published:** 2024-06-20

**Authors:** Hannah Musco, Kate Beecher, Kirat K. Chand, Roslyn N. Boyd, Paul B. Colditz, Julie A. Wixey

**Affiliations:** ^1^ UQ Centre for Clinical Research, Faculty of Medicine, The University of Queensland, Brisbane, QLD, Australia; ^2^ Queensland Cerebral Palsy and Rehabilitation Research Centre, Child Health Research Centre, Faculty of Medicine, The University of Queensland, Brisbane, QLD, Australia; ^3^ Perinatal Research Centre, Royal Brisbane and Women’s Hospital, Brisbane, QLD, Australia

**Keywords:** fetal growth retardation, intrauterine growth restriction, small for gestational age, detection, newborn, brain

## Abstract

Fetal growth restriction (FGR) impacts 5%–10% of pregnancies and is associated with increased risk of mortality and morbidity. Although adverse neurodevelopmental outcomes are observed in up to 50% of FGR infants, a diagnosis of FGR does not indicate the level of risk for an individual infant and these infants are not routinely followed up to assess neurodevelopmental outcomes. Identifying FGR infants at increased risk of adverse neurodevelopmental outcomes would greatly assist in providing appropriate support and interventions earlier, resulting in improved outcomes. However, current methods to detect brain injury around the time of birth lack the sensitivity required to detect the more subtle alterations associated with FGR. Blood biomarkers have this potential. This systematic review assessed the current literature on blood biomarkers for identifying FGR infants at increased risk of adverse neurodevelopmental outcomes at >12 months after birth. Four databases were searched from inception to 22 February 2024. Articles were assessed for meeting the inclusion criteria by two reviewers. The quality of the included article was assessed using Quality Assessment of Diagnostic Accuracy Studies-2. A summary of findings is presented as insufficient articles were identified for meta-analysis. Excluding duplicates, 1,368 records were screened with only 9 articles considered for full text review. Only one article met all the inclusion criteria. Quality assessment indicated low risk of bias. Both blood biomarkers investigated in this study, neuron specific enolase and S100B, demonstrated inverse relationships with neurodevelopmental assessments at 2 years. Four studies did not meet all the inclusion criteria yet identified promising findings for metabolites and cytokines which are discussed here. These findings support the need for further research and highlight the potential for blood biomarkers to predict adverse outcomes.

**Systematic Review Registration:**

https://www.crd.york.ac.uk/prospero/display_record.php?RecordID=369242, Identifier CRD42022369242.

## 1 Introduction

Fetal growth restriction (FGR) is a major cause of increased risk of mortality and morbidity in infants occurring in 5%–10% of pregnancies with higher rates in low-income countries (1, 2). FGR is defined as ultrasound estimated fetal weight below 10th percentile with other factors included for diagnosis such as umbilical artery Doppler flow assessment, physiological determinants and neonatal features of malnutrition (3–5).

The causes of FGR are complex and can arise from several potential sources including maternal (hypertension, diabetes, preeclampsia, malnutrition, maternal age) (6), placental (placental dysfunction leading to poor blood supply and nutrient transfer) (7), and fetal (multiple pregnancy, congenital infections, genetics) (6). Placental insufficiency is the most common cause where a reduction in supply of oxygen and nutrients to the developing fetus results in a chronic hypoxic environment impacting normal growth and development of the fetus.

The chronic hypoxic environment that FGR fetuses are exposed to *in utero* particularly affects brain development. Lower grey matter and white matter complexity are major neuropathophysiological features of FGR which are shown to persist after birth (8). Changes in white matter microstructure are observed at 8–10 years of age in FGR compared to normally grown (NG) children (9). Adverse long-term neurological outcomes are reported in 24%–53% of FGR infants (10, 11), including language delays, learning and behavioral problems and cerebral palsy (CP) (12–14). Yet, the ability to identify FGR neonates at risk of adverse neurodevelopmental outcomes is lacking. Unlike for neonatal hypoxic-ischemic encephalopathy (HIE) where severity of injury is assessed using the Sarnat Grading Scale, there is no defined criteria at birth to score injury in FGR babies. Therefore, the likelihood to predict which newborn may have adverse outcomes is not clear and therefore interventions and support systems are commonly not implemented until developmental delays become evident years later. Due to evidence of positive impacts on CP infants with early physiotherapy intervention after birth (15), it is crucial to determine effective and efficient methods to identify FGR infants at risk of long-term adverse neurodevelopmental outcomes.

Studies have investigated the reliability of cranial ultrasounds, magnetic resonance imaging (MRI) and electroencephalography (EEG) to provide an indication of risk of adverse neurodevelopmental outcomes for FGR infants (16, 17). All three methods however, require further evidence for confirming the sensitivity of predicting long-term adverse neurodevelopmental outcomes in FGR. Cranial ultrasounds can identify brain structure abnormalities such as brain volume neuropathologies including intracranial hemorrhages and hydrocephalus (18). Although MRIs can detect more subtle structural changes such as white matter injury which has associations with cognitive and motor outcomes, the costs, resources and expertise required do not make it an ideal method for widespread implementation (19, 20). EEG studies have shown promise in identifying FGR infants at risk of adverse neurodevelopmental outcomes, however studies to date have utilized only visual interpretation of EEGs which relies on operator proficiency (17), rather than objective quantitative analysis.

Due to the limitations of these current methods for measuring subtle brain alterations, the search for sensitive predictors of long-term adverse neurological outcomes is ongoing. Blood biomarkers have the potential to provide an objective indication of risk of adverse brain outcomes. The diagnostic and prognostic utility of blood biomarkers to detect adverse brain outcomes is currently being investigated in adult and neonatal neurological disease states. There is evidence to suggest that alterations in the brain due to injury or disease are reflected in the blood. For example, alterations in proteins have been detected in blood samples from amyotrophic lateral sclerosis patients that distinguish these patients from healthy controls and other brain conditions (21–23). Furthermore, alterations in proteins present in blood of traumatic brain injury patients are correlated with adverse brain outcomes (24–26). Interest in blood biomarkers has grown with several recent studies examining blood biomarkers in FGR neonates. This systematic review examines the current literature to identify potential blood biomarkers that indicate risk of long-term adverse neurodevelopmental outcomes in FGR neonates with the ultimate goal to enable earlier interventions. We hypothesize blood biomarkers may indicate risk of adverse neurodevelopmental outcomes in FGR infants shortly after birth.

## 2 Methods

### 2.1 Search strategy

This review was registered with the International Prospective Register of Systematic Reviews (PROSPERO) November 2022 (ID: CRD42022369242) (27) and designed in accordance with the PRISMA Guidelines.

A comprehensive search was undertaken of online databases Pubmed, Cumulative Index to Nursing and Allied Health Literature (CINAHL), Web of Science and Excerpta Medica Database (EMBASE) from inception to 22 February 2024. A PICO (patient/population, intervention, comparison and outcomes) search strategy was implemented using Medical Subject Headings (MeSH) and keywords for the target population of “fetal growth restriction infants” with outcomes of “brain injury” or “neurological outcomes”. Additional search terms (listed in Appendix A) were added to identify studies with “blood biomarkers”. The search was limited to “humans” and only articles with professional English translations available were considered. Likelihood of language bias was considered to be minimal (28, 29).

### 2.2 Inclusion/exclusion criteria

No restrictions on language were used when conducting searches. Studies were eligible for inclusion if they were conducted in humans and in which all the following criteria were met:

• The participant population was defined as infants born <10th percentile for gestational age and at least one other clinical indication used for diagnosis of FGR such as umbilical artery Doppler assessment, physiological determinants and neonatal features of malnutrition as described in the current literature (3–5).
• The same protocol was conducted in a cohort of NG infants (birthweight >10th percentile) with no other clinical indications of FGR, low birthweight, malnutrition or major congenital abnormalities.
• An applicable, valid, reliable and standardised reference test was used to identify cognitive and/or motor delays at least 12 months after birth such as Bayley Scales of Infant and Toddler Development (BSITD) or school age outcomes.

The inclusion criteria for long-term adverse neurodevelopmental outcomes were intended to ensure that identified articles provided long-term follow-up rather than identifying brain abnormalities at birth. This was crucial to achieve the aim of this review, given that current brain injury detection methods such as cranial ultrasounds, MRIs or EEGs do not detect the subtle brain alterations associated with FGR adverse outcomes as discussed above. Studies were excluded if they were commentaries, cross sectional or descriptive studies or single case reports.

### 2.3 Data extraction and quality assessment

The search strategy was jointly devised by HM, KB and RB. Titles and abstracts were extracted and screened by HM and KB, supervised by JAW. The full text of all articles deemed potentially eligible were reviewed and assessed by HM and KB for conformity to all inclusion criteria. Conflicting viewpoints were discussed until consensus was reached or resolved by JAW.

A single study that met the inclusion criteria was extracted for further assessment. The quality, risk of bias and applicability of patient selection of the study was assessed using the revised Quality Assessment of Diagnostic Accuracy Studies (QUADAS-2) by HM and KB (30). Insufficient articles met the inclusion criteria for meta-analysis to be conducted.

## 3 Results

**Figure 1:**
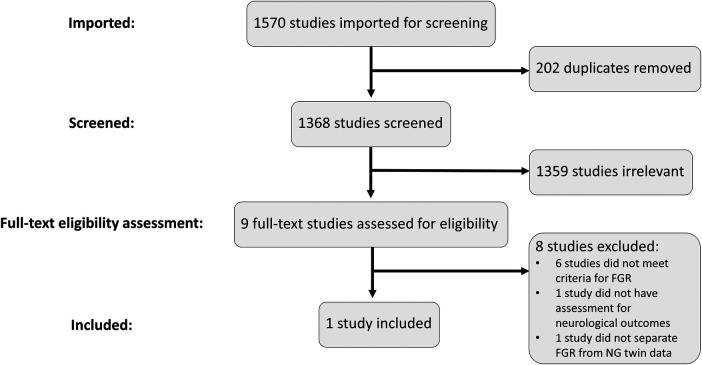
PRISMA flowchart and selection process. Studies were imported into Covidence to conduct the screening and eligibility assessment processes. Duplicates were removed, leaving 1,368 studies to be screened. Relevant studies were included for full-text review where the eligibility criteria were assessed. Only one study met all the inclusion criteria and was included for further analysis.

Excluding duplicates, 1,368 records were retrieved across the four databases searched (Figure 1). Only nine studies remained for full-text review after the initial title and abstract screening (Figure 1). Five of these studies did not include a control group of NG infants (31–35). These would have also been excluded for other reasons such as not meeting FGR definition (very low birth weight, preterm birth only), not assessing neurodevelopmental outcomes, or that no blood biomarkers were assessed. One study was excluded as it did not include neurodevelopmental assessments (36) and another was excluded as it reported only the presence of brain injury for the FGR cohort (37). Finally, one study was a twin study but was excluded as it did not separate the FGR twins from the normally grown pair for comparison (38).

This resulted in only one remaining study to be included for analysis.

To broaden our perspective, we considered whether any additional papers would be included if we altered our inclusion criteria to include small for gestational age (SGA) and FGR. Small for gestational age (SGA) differs from FGR in that it is solely defined by birthweight <10th percentile (3–5). We observed, however, that the studies included for full text review that included SGA cohorts either did not have a cohort of NG infants as controls or did not conduct a neurodevelopmental assessment >12 months of age for the NG cohort (31–37). Therefore, even when broadening the inclusion criteria to include SGA infants, our search of the current literature still only yielded one study that met all inclusion criteria.

Thirty-one FGR and 25 non-FGR or NG neonates were recruited for this study (39). FGR neonates were recruited consecutively, and NG neonates were gestational age-matched (±7 days) to the FGR neonates. FGR was confirmed after birth if birthweight <3rd percentile or, <10th percentile with abnormal Doppler study or abnormal cerebroplacental ratio. Mean gestational age at delivery was not statistically significantly different between NG and FGR neonates (262 days [standard deviation (SD) = 21] and 257 days (SD = 15) respectively). Gender differences were not reported. Birthweight and birthweight percentile were much lower in the FGR cohort compared to NG neonates as expected. Incidence of nulliparity, preeclampsia and severe preeclampsia were all significantly higher in the FGR cohort.

Both fetal umbilical arterial and venous blood samples were collected at the time of delivery and supernatant stored at −80°C (39). Serum S100B protein and neuron specific enolase (NSE) assays were performed to measure the protein levels in both sample types. These formed the four individual index tests examined in this study. At 2 years of age, developmental function was assessed using BSITD third edition (BSITD III), conducted as per the test manual.

### 3.1 Methodological quality of included study with QUADAS-2

#### 3.1.1 Participant selection

The included study was considered to have a low risk of bias for the selection of participants. FGR singleton pregnancies were consecutively enrolled in the study with age-matched controls (39). Reasons for exclusion from the study included maternal illicit drug use, maternal endocrine pathologies, fetal infection, fetal malformations, genetic abnormalities, failure to follow clinical protocols or delivery in a hospital not included in the study. It was important to exclude cases where there may have been adverse neurodevelopmental outcomes due to other causes and therefore, these reasons for exclusion were reasonable.

There was also low concern regarding the applicability of the study. Included FGR participants were confirmed using three definitions of FGR consistent with Australian clinical standards and the inclusion and exclusion criteria matched the aims of this review.

#### 3.1.2 Index tests

Mazarico, Llurba (39) included four index tests: S100B and NSE levels each measured in maternal venous blood and fetal umbilical arterial blood. All these tests were rated as low risk of bias as the measurement of S100B and NSE in the blood samples were conducted objectively. Concerns regarding the applicability of these tests was also considered to be low. Concentrations of S100B and NSE in both maternal venous blood and fetal umbilical arterial blood were compared with the results of the neurodevelopmental assessment conducted at 2 years corrected age to identify potential relationships between results of these tests.

#### 3.1.3 Reference standard

BSITD III was the reference standard used by Mazarico, Llurba (39) to determine neurodevelopmental outcomes for the study participants. As a formal assessment tool to identify developmental delays in early childhood (40), BSITD III is regularly used in the literature to assess development at 2 years of age. As is standard procedure, BSITD III was performed by a trained psychologist without knowledge of the study group and the perinatal outcomes for each participant. Some aspects of the BSITD III involved parent-report questionnaires which are also standard for conducting the BSITD III. Considering this, the reference test used was considered to have a low risk of bias and there were low concerns regarding the applicability of the study.

#### 3.1.4 Flow and timing

There were also low concerns for the risk of bias for the flow and timing of the study. All patients received the same reference standard and were included in the analysis. Although there was a delay of 2 years before BSITD III was conducted, this was expected and consistent with the design of this study.

### 3.2 Relationships between S100B or NSE concentrations and adverse neurodevelopmental outcomes

Table 1 summarizes the findings from Mazarico, Llurba (39). Each subtest of BSITD III was investigated for associations with NSE or S100B levels in the blood samples collected. For FGR infants, there was a statistically significant inverse relationship between concentration of S100B in maternal serum and the adaptive behavior test. However, statistically significant inverse relationships were also observed for NG infants between the concentration of S100B in cord blood and the language composite score values. For NG infants, the concentration of NSE in cord blood was also in a statistically significant inverse relationship with each of the results of the fine motor subtest and the social-emotional test.

**Table 1:** Summary of significant relationships of investigated blood biomarkers from Mazarico, Llurba (39) in identified participants that are associated with neurodevelopmental outcomes at 2 years of age.

Biomarker	Sample	Cohort	Statistically significant inverse relationship with
S100B	Maternal serum	All participants	–
		FGR	Adaptive behaviour test
		Non-FGR	–
	Umbilical cord blood	All participants	Cognitive test and expressive communication subtest.
		FGR	–
		Non-FGR	Language composite score values.
NSE	Maternal serum	All participants	–
		FGR	–
		Non-FGR	–
	Umbilical cord blood	All participants	Cognitive test, fine motor subtest and social-emotional test.
		FGR	–
		Non-FGR	Fine motor subtest and social-emotional test.

Mazarico, Llurba (39) found no differences between the FGR and NG groups for the results of any subtests of BSITD III. This indicated that outcomes were similar across groups. Across all infants, the concentration of S100B in cord blood displayed a statistically significant inverse relationship with the results of each the cognitive and expressive communication tests (39). Statistically significant inverse relationships were also observed for all infants between NSE concentrations in cord blood and the results for each of the cognitive test, fine motor subtest and social-emotional test (39).

## 4 Discussion

This is the first systematic review to our knowledge of potential blood biomarkers for identifying risk of adverse neurodevelopmental outcomes in FGR. Only one study was identified as relevant to this systematic review despite ensuring our search terms were sufficiently broad to account for differences in terminology in the literature. This systematic review has the scientific merit of our search strategy returning key articles to inform a scientifically robust examination of the topic. If inclusion criteria were broadened more articles may be included, but this would negatively impact on scientific quality and impact as meta-analysis could not be conducted.

This search of the literature highlighted a number of gaps, limitations and differences in terminology that exist. Firstly, the search terms used were relatively broad to ensure these captured all potential publications investigating blood biomarkers for detecting risk of adverse neurodevelopmental outcomes in FGR infants. For example, some studies use “intrauterine growth restriction” (IUGR) to describe FGR (39), while other studies report SGA but also include cohorts that matched our definition of FGR (37). Another example of this was sufficiently capturing a variety of possible descriptions for “adverse neurodevelopmental outcomes”. Some articles investigated “neural injury” (39), while others investigated “brain injury” (34), or “white matter damage” (35). Although a number of different search terms were used as alternatives to broaden the search query, only nine publications were included in the full-text review, with only one meeting all inclusion criteria.

Only two blood biomarkers were considered in the identified study (39) which was deemed high quality using QUADAS-2. These results indicate the potential blood biomarkers have for indicating risk of adverse neurodevelopmental outcomes for FGR infants. However, as eight studies were excluded from this systematic review for multiple reasons, as mentioned above, this demonstrates the clinical literature lacks rigor. Preclinical studies are extremely important to answer research hypotheses as they can be undertaken in controlled environments. However, changing the approach of this review to include preclinical literature is not feasible as there are no studies to our knowledge that investigate blood biomarkers of brain injury in animal models of FGR. This review identifies the need for future studies to further investigate blood biomarkers for risk of adverse neurodevelopmental outcomes in FGR infants.

Neurodevelopmental outcomes measured at 2 years were not statistically significantly different between NG and FGR infants in the identified study (39). This finding does not align with the majority of studies demonstrating significant adverse long-term neurodevelopmental outcomes observed in FGR compared with NG infants (10–14). This may be due to the selection process for the NG group. What has been described here as the NG control group for Mazarico, Llurba (39) is more appropriately described in the study as a non-FGR group. Although this group is not classified as FGR, these infants have comorbidities such as 8% from preeclamptic pregnancies, 16% with respiratory distress syndrome, 28% neonatal morbidity and 32% neonatal admission (compared to 24%, 25.8%, 32.2% and 58% for FGR respectively) (39). The results indicate that the blood levels of NSE and S100B may indicate risk of adverse neurodevelopmental outcomes for all infants. This is an interesting and important finding, but also suggests a significant limitation of the study, that the non-FGR group also potentially has comparable rates of adverse neurodevelopmental outcomes and may not be a true representation of NG infants. Furthermore, a recent systematic review demonstrates the potential for NSE and S100B as biomarkers of injury in the newborn. They showed levels of both biomarkers correlate with unfavorable outcomes in HIE newborns (41). Although the paper concluded more studies are required to determine the sensitivity and specificity of these biomarkers, this further demonstrates the potential for these two biomarkers to predict adverse outcomes.

Four studies were excluded from the systematic review due to not meeting all the selection criteria, however some of their findings were of particular interest and may suggest other blood biomarkers to examine in future long-term studies.

The first of these studies considered correlations between cord blood gas and outcomes at 6–12 months in infants with low birthweight (31). Although the best single predictor for mental development index was pH of infant arterial blood gas, the best single predictor for psychomotor development index was gestational age (31). These findings demonstrate the need for research into blood biomarkers as indicators of risk of adverse outcomes to be broadened as current measures such as cord blood gases provide only limited insight.

Another study excluded after full-text review compared blood biomarker levels in FGR infants with brain injury to infants without brain injury on postnatal days 1–5 (34). Although increases in interleukin (IL)-6, IL-8, IL-10 and glial fibrillary acidic protein (GFAP) were observed in FGR infants with brain injury (34), it is not clear whether similar increases would be observed in cases of more subtle brain injury in FGR infants that are not currently detectable at birth.

A third study excluded from full text review identified increases in levels of NSE and ischemia-modified albumin (IMA) for SGA infants with brain abnormalities compared to SGA infants without brain abnormalities (37). These brain abnormalities were identified using cranial ultrasounds 12 h after birth (37). Although this finding is interesting, like the previous studies highlighted, it also does not provide evidence on whether these proteins indicate risk for adverse long-term outcomes in FGR infants.

Finally, a twin study where one twin was FGR and the other >25th percentile for birthweight, was ultimately excluded due to the NG sibling's data being combined with the FGR sibling (38). Correlations were identified between maternal metabolite levels of creatine, L-serine, L-arginine, and L-histidine with communication, first walking time, gross and fine motor function, and problem solving (38). With these findings indicating correlations between maternal metabolite levels and outcomes at 2 years of age even when FGR only impacts one twin, this demonstrates the potential for biomarkers to provide an indication of risk of adverse outcomes at birth.

The current literature identifies inverse relationships between blood biomarkers, NSE and S100B, measured at birth and neurodevelopmental outcomes at two years of age (39). This systematic review highlights the need for more research to determine blood biomarkers that indicate risk of adverse neurodevelopmental outcomes in FGR infants. Future studies assessing outcomes at school age would also be beneficial as cognitive and motor impairments are commonly observed at this age range, but unfortunately no FGR blood biomarker studies have assessed school age outcomes. Although there are many challenges for future research in this area, identifying blood biomarkers that indicate risk of adverse neurodevelopmental outcomes for FGR infants has the potential to vastly improve the lives of these infants, as up to 50% of FGR infants are reported to have adverse neurodevelopmental outcomes (10, 11). With notable improvements observed in infants with CP when interventions are implemented earlier (15), it is crucial to investigate effective and efficient methods to identify FGR infants at risk of long-term adverse neurodevelopmental outcomes in order to implement earlier support and interventions. This will enable these infants to have the best possible long-term neurodevelopmental outcomes.

## Data Availability

The original contributions presented in the study are included in the article/Supplementary Material, further inquiries can be directed to the corresponding author.
